# Bond–Slip Relationship for CFRP Sheets Externally Bonded to Concrete under Cyclic Loading

**DOI:** 10.3390/ma11030336

**Published:** 2018-02-26

**Authors:** Ke Li, Shuangyin Cao, Yue Yang, Juntao Zhu

**Affiliations:** 1 Department of Civil Engineering, Zhengzhou University, Zhengzhou 450001, China; irwinlike@163.com; 2 Department of Civil Engineering, Tsinghua University, Beijing 100084, China; 3 Department of Civil Engineering, Southeast University, Nanjing 210096, China; 101000873@seu.edu.cn; 4 Department of Transportation Science and Engineering, Beihang University, Beijing 100191, China

**Keywords:** carbon fiber-reinforced polymer, concrete, interface, bond–slip model, cyclic loading

## Abstract

The objective of this paper was to explore the bond–slip relationship between carbon fiber-reinforced polymer (CFRP) sheets and concrete under cyclic loading through experimental and analytical approaches. Modified beam tests were performed in order to gain insight into the bond–slip relationship under static and cyclic loading. The test variables are the CFRP-to-concrete width ratio, and the bond length of the CFRP sheets. An analysis of the test results in this paper and existing test results indicated that the slope of the ascending segment of the bond–slip curve decreased with an increase in the number of load cycles, but the slip corresponding to the maximum shear stress was almost invariable as the number of load cycles increased. In addition, the rate of reduction in the slope of the ascending range of the bond–slip curve during cyclic loading decreased as the concrete strength increased, and increased as the load level or CFRP-to-concrete width ratio enhanced. However, these were not affected by variations in bond length if the residual bond length was longer than the effective bond length. A bilinear bond–slip model for CFRP sheets that are externally bonded to concrete under cyclic loading, which considered the effects of the cyclic load level, concrete strength, and CFRP-to-concrete ratio, was developed based on the existing static bond–slip model. The accuracy of this proposed model was verified by a comparison between this proposed model and test results.

## 1. Introduction

During the last few decades, significant progress has been made in the promotion and application of carbon fiber-reinforced polymer (CFRP) composites in retrofitting and strengthening civil constructions due to their advantages, which are namely, light weight, high strength, excellent fatigue resistance, and a simple application method [1]. Especially for reinforced concrete (RC) structures, externally bonded CFRP sheets/plates have been widely used as a valid reinforcement method [2]. A good interfacial bonding property between CFRP and concrete provides an important precondition for the excellent work performance of this reinforcement method. Many CFRP-strengthened structures, such as RC bridges, are required to suffer both static and fatigue loading during the service life. So, a precise expression of bond–slip relationship of the CFRP–concrete interface under static and fatigue loading is a base for the reasonable and accurate prediction of the mechanical performance of RC bridges that are strengthened by externally bonded CFRP.

Systematic investigations have been carried out on the bond–slip behavior between CFRP and concrete under quasi-static loading. Comprehensive research studies, which include the mechanical experiments [3,4,5,6,7], analytical analysis, and numerical analysis [8,9,10,11,12,13,14,15,16], have been performed on static bond–slip performance. The test methods that are popularly adopted to investigate interface bond included beam tests and direct pull-out tests (which comprised double-shear pull-out tests and singer-shear pull-out tests). Various static bond–slip models, which were applied to a CFRP–concrete interface under different working conditions, were proposed by researchers [6,7,14,15,16]. A rise phase followed by a fall phase was commonly incorporated in the proposed accurate or simplified models [6,7,14,15,16,17]. The mechanical indicators of CFRP–concrete interface, namely, the maximum bond shear stress and its corresponding local slip—the local slip when bond shear stress decreased to zero and interfacial fracture energy—were generally considered as the main model parameters [6,7,14,15,16,17]. The prime influencing factors of the bond characteristic of CFRP externally bonded to concrete involved concrete strength, CFRP bond length, the CFRP-to-concrete width ratio, temperature, the properties of the adhesive layer, and external compression applied on the interface [3,4,5,15,16].

Compared with thorough research studies on the static bond properties of the CFRP–concrete interface, limited studies have been conducted on the interfacial bond behavior under fatigue loading. The available experimental and theoretical studies involved in bond fatigue performance are mainly focused on the issues of fatigue life prediction [18,19,20], fatigue crack propagation behavior [20,21,22,23], and fatigue bond–slip performance [24,25,26,27,28,29,30]. Existing research studies have revealed that fatigue loading would produce degradation in the bond properties of a CFRP–concrete interface, which should be noted in the fatigue design of externally bonded CFRP systems [24,25,26,27,28,29,30]. Ko et al. [27] conducted double-shear pull-out tests (taking sheet layers and loading hysteresis as test variables) to explore the bond stress–slip relationship between CFRP sheets and concrete under cyclic loading, and proposed a bond stress–slip model for the interface under cyclic loading, which considered seven empirical parameters; namely, the maximum bond shear stress and its corresponding slip, the characteristic constant of bond–slip curve, the unloading stiffness, the ultimate slip, the friction stress, and the negative friction stress. Daud et al. carried out 24 single-shear pull-out tests [28] to study the fatigue behavior of the bond–slip between CFRP plates and concrete. The test results indicated that cyclic loading caused the reduction in both the maximum bond strength and the fracture energy of the CFRP–concrete interface, and the amount of reduction depended on the stiffness of the CFRP plate, rather than the concrete strength. Zhang [29] developed a bond stress–slip model (which was expressed as a function of the loading/unloading linear slip, the stiffness of the unloading branch in slip, and the number of fatigue load cycles) for CFRP plates that were externally bonded to concrete under fatigue loading based on double-lap pull-out test results. Zhu et al. [30] carried out beam tests in order to investigate the fatigue behavior of the CFRP–concrete interface bond, considering the effects of loading amplitude and concrete strength, and developed a nonlinear bond–slip model for CFRP sheets that are bonded to concrete under fatigue loading based on test results.

Although great effects have been devoted to explore the static bond–slip relationship of the CFRP–concrete interface, the available studies [24,25,26,27,28,29,30] on the bond–slip relationship of CFRP composites that are externally bonded to concrete under fatigue loading are scarce. The influence factors of the bond–slip relationship of an externally bonded CFRP system have not been fully investigated. Therefore, in this paper, the bond–slip relationship between CFRP sheets and concrete were studied experimentally and analytically, considering the effects of cyclic load level, concrete strength, the CFRP-to-concrete width ratio and the bond length of CFRP sheets. The main works involved in this study were: (a) static and cyclic loading tests (using modified beam specimens) taking the factors, namely, the CFRP-to-concrete width ratio and the bond length of the CFRP sheets, as test variables to evaluate the bond–slip relationship of the CFRP sheets bonded to concrete; (b) exploring the laws of influence of the factors that affect the bond–slip relationship under cyclic loading; and (c) developing a bilinear bond–slip relationship of CFRP sheets externally bonded to concrete under cyclic loading, considering the effects of the cyclic load level, concrete strength, and CFRP-to-concrete width ratio.

## 2. Experimental Study

### 2.1. Experimental Program

Modified beam tests were performed in order to investigate the fatigue behavior of bond stress–slip of the CFRP–concrete interface in reinforced concrete beams strengthened with CFRP sheets. The variables, which included a CFRP-to-concrete width ratio and the bond length of CFRP sheets, were taken into account in the test reported in this paper. Two groups of specimens (defined by C and D groups, respectively) were designed considering the variables of the CFRP-to-concrete width ratio and the bond length of CFRP sheets, respectively. In addition, another modified beam test taking loading amplitude and concrete strength as test variables (which included two groups of specimens defined by A and B, respectively) has been conducted by our research group [30]. The bond length of the CFRP sheet of each specimen was selected to be longer than the effective bond length (which was calculated referring to Chen and Teng [31]). Each specimen in groups A, B, C, and D is detailed in Table 1.

**Figure 1:**
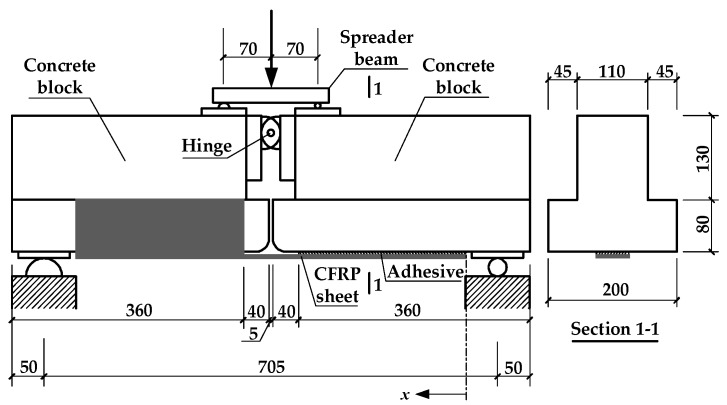
Schematic of the test setup (unit: mm).

**Figure 2:**
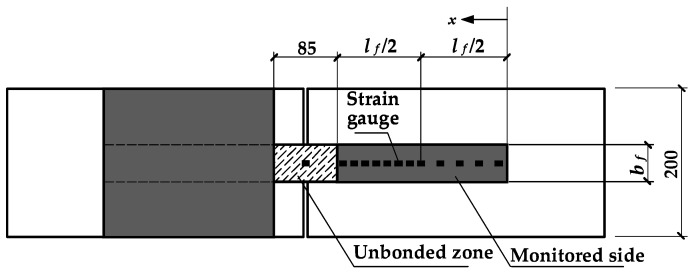
Locations of strain gauges (unit: mm).

The modified beam specimen used in this test is displayed in Figure 1. It can be seen in Figure 1 that as a part of the beam, a steel hinge was applied to connect two identical concrete blocks at the top, while two layers of CFRP sheets were bonded to the bottom of the two concrete blocks. In addition, one concrete block was strengthened by a layer of lateral CFRP sheet in order to make failure occur at the bottom of the other concrete block (the monitored side shown in Figure 2). The elastic modulus and tensile strength of the CFRP sheets used in this test (with a nominal thickness of 0.111 mm) were tested to be 225 GPa and 4148 MPa, respectively. The tensile strength and elastic modulus of the epoxy resin that was used to bond the CFRP sheets were 47.2 and 2857.7 MPa, respectively, which were provided by the manufacturer. The test result of the average 28-day cube strength of concrete, *f_cu_*, the CFRP-to-concrete width ratio, *b_f_*/*b_c_* (in which *b_f_* indicates the width of the CFRP sheets, and *b_c_* indicates the width of the concrete surface), and the bond length of the CFRP sheets at the monitored side, *l_f_*, for each specimen are listed in Table 1.

Both quasi-static loading and cyclic loading tests were conducted on specimens in each group by using the loading form of four-point bending (seen in Figure 1). All of the quasi-static loading tests were performed at a rate of 0.001 mm/s until failure to obtain the static load that was applied on the spreader beam and corresponded to the debonding of the CFRP sheets (*P_u_*) (which is referred to as the debonding load below in this paper) of the specimens in each group. A constant-amplitude sinusoidal loading with the lower bound value (*P*_min_) of 15% of the debonding load (*P_u_*) was applied on the spreader beam of each cyclic specimen at a frequency of 2.5 Hz. The upper bound value of the cyclic loading that was applied on the spreader beam, *P*_max_, the loading amplitude, ∆*P* (which is equal to the difference between *P*_max_ and *P*_min_), and the ratio of the loading amplitude to the debonding load, ∆*S*, for each cyclic specimen, are listed in Table 1. The *P*_max_ of each static specimen listed in Table 1 was equal to the debonding load (*P_u_*) of the static specimen. In the static and cyclic loading tests, strain gauges were used to monitor the evolution of the strains in the CFRP sheets. The strain gauges were placed on the surface of the CFRP sheets along the centerline in the monitored zone. A spacing of 10 mm was adopted between the continuous strain gauges on the CFRP sheet on a half of the bonded region near the midspan. The spacing between each of the two continuous strain gauges on the other half of the bonded region near the free end was set to be 15 and 30 mm for the specimens with bond lengths of 160 and 240 mm, respectively.

### 2.2. Experimental Results and Discussion

#### 2.2.1. Failure Modes

**Figure 3:**
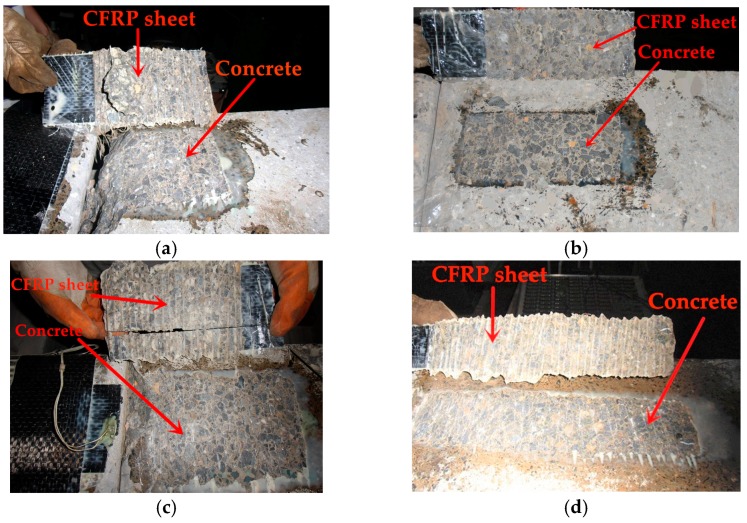
The failure modes of specimens: (**a**) C-02; (**b**) C-1; (**c**) C-2; and (**d**) D-1.

Both the static and cyclic test specimens collapsed due to the debonding of the CFRP sheets along the CFRP–concrete interface. The failure modes of specimens in groups A and B have been presented and discussed by Zhu et al. [30]. Figure 3a shows the typical failure mode of the static specimens in groups C and D. Figure 3b–d give the failure modes of the cyclic specimens in groups C and D. It can be detected from Figure 3 that a thin layer of concrete was peeled off by the CFRP sheet, and the width of this thin layer of concrete was slightly larger than the width of the CFRP sheet. This phenomenon revealed that the debonding propagated in the concrete layer near the CFRP–concrete interface for the specimens under both static loading and cyclic loading. The number of cycles at failure (*N*) for each specimen is summarized in Table 1. A comparison of the test results of specimens A-2, C-1, and C-2 indicates that the numbers of cycles at failure increased as the CFRP-to-concrete width ratio decreased. The reason for this phenomenon may be that the static bond strength of the CFRP–concrete interface (which was defined as the tensile stress of the CFRP sheet when debonding between the CFRP sheet and concrete occurred under static loading) increased as the CFRP-to-concrete width ratio decreased [15], which would enhance the fatigue resistance of the CFRP–concrete interface. A comparison of the test results of specimens A-0 and D-0 showed that the debonding load was almost unchanged with the increase in the bond length if the bond length is longer than the effective bond length, which was also found by Lu et al. [15]. Whereas, a comparison of the test results of specimens A-2 and D-1 indicated that the numbers of cycles at failure increased as the bond length increased, because the increased bond length will need an increased number of cycles to fail.

#### 2.2.2. Bond Stress–Slip Relationship

The bond shear stress between the CFRP sheet and concrete can be captured according to the CFRP-sheet strains, as measured by the strain gauges that were bonded to the surface of the CFRP sheet. The average interfacial bond shear stress of the region between two adjacent measuring points (*τ*) can be calculated by the following equation:

(1) $$\tau =t_{f}\frac{d\sigma }{dx}=t_{f}E_{f}\frac{d\varepsilon }{dx}$$

where *t_f_* and *E_f_* indicate the thickness and elastic modulus of the CFRP sheet, respectively, d*σ* denotes the difference in the tensile stress between two adjacent measuring points, d*ε* is the strain difference between two adjacent measuring points, and d*x* represents the distance between two adjacent measuring points. The average bond shear stress of the CFRP–concrete interface between two adjacent measuring points calculated by Equation (1) can be seen as an approximation of the interfacial bond shear stress at the midpoint of the region between two adjacent measuring points.

The local slip (*S*) at a certain point (which is the distance of *x_i_* from the free end of the CFRP sheet) can be calculated by integrating the strains of the CFRP sheet from the free end to the certain point, which is shown as:

(2) $$S=\int_{0}^{x_{i}}\varepsilon dx$$

The bond–slip relationship at a certain location (which is chosen to be about 50 mm away from the bond end of the CFRP sheet near the midspan) of the CFRP–concrete interface at a different number of load cycles for each specimen can be obtained by processing the test results using Equations (1) and (2). When the difference between the strain readings of the two adjacent stain gauges—which were considered to calculate bond shear stress—reached the peak value, the corresponding number of load cycles was set to be one, which is seen as the starting point to count load cycles while plotting the bond–slip curve.

**Figure 4:**
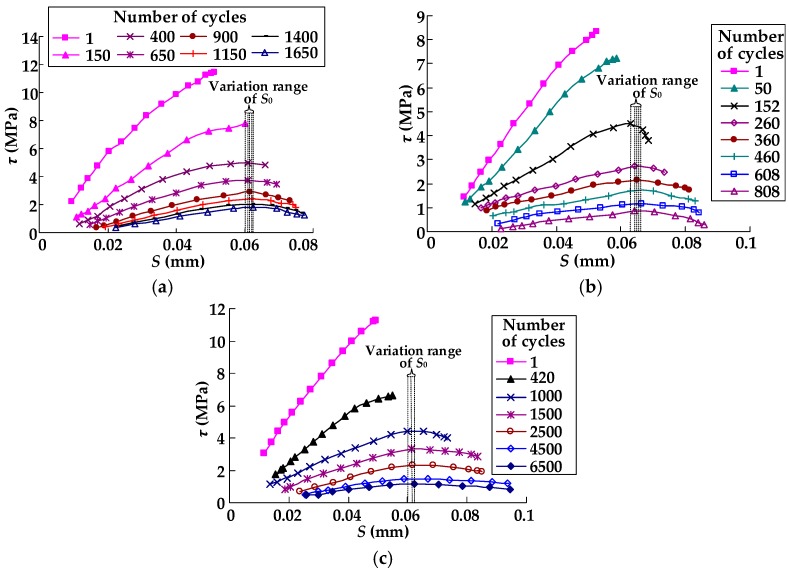
Bond–slip curves at different load cycles for specimens: (**a**) C-1; (**b**) C-2; and (**c**) D-1.

Zhu et al. presented the bond–slip curves at different number of load cycles for cyclic specimens in groups A and B [30]. The bond–slip curves at different number of load cycles for cyclic specimens in groups C and D are shown in Figure 4. It can be seen from Figure 4 that the bond–slip curve degraded continuously as the number of load cycles increased. Specifically, for the bond–slip curves at different load cycles, the overall slope of the ascending portion (before the shear stress reach the peak value) decreased as the number of load cycles grew. In addition, the maximum bond shear stress (*τ*_max_) decreased with an increase in the number of load cycles for each specimen. The slip corresponding to the maximum bond shear stress (*S*_0_) increased slightly with an increasing number of load cycles. As shown in Figure 4, the range of variation in *S*_0_ with the number of load cycles was so small that *S*_0_ can be seen to be unchanged during the cyclic loading test. The same phenomenon can be observed in the bond–slip curves at a different number of load cycles for the cyclic specimens in groups A and B, which was presented by Zhu et al. [30].

## 3. Analysis of the Bond–Slip Model

### 3.1. Verification of the Existing Bond–Slip Model under Static Loading

Lu et al. provided a bilinear bond–slip model for the CFRP–concrete interface under static loading [15]:

(3) $$\left\{ \begin{array}{cc} \tau =\tau_{\max}\frac{S}{S_{0}} & S\leq S_{0}\\ \tau =\tau_{\max}\frac{S_{f}-S}{S_{f}-S_{0}} & S_{0}<S\leq S_{f}\\ \tau =0 & S>S_{f} \end{array}\right.$$

where *τ*_max_ is the maximum local shear stress, *S*_0_ is the local slip corresponding to *τ*_max_, and *S_f_* denotes the value that the local slip reaches when the local shear stress just decreased to 0. The parameters of this bond–slip model can be expressed as follows, according to our previous work [30]:

(4) $$\tau_{\max}=\beta_{w}(0.2233f_{cu}-2.1433)$$

(5) $$S_{0}=0.0195\beta_{w}f_{t}\leq 0.06$$

(6) $$S_{f}=2G_{f}/\tau_{max}$$

where:

(7) $$\beta_{w}=\sqrt{\frac{2.25-b_{f}/b_{c}}{1.25+b_{f}/b_{c}}}$$

(8) $$G_{f}=\beta_{w}^{2}(0.029f_{cu}-0.2668)$$

In Equations (4)–(8), *f_cu_* and *f_t_* are the cube compressive strength and the tensile strength of the concrete, respectively, *β*_w_ is the coefficient considering the effect of the CFRP-to-concrete width ratio, *G_f_* is the interfacial fracture energy, *b_f_* is the width of the CFRP sheets, and *b_c_* is the width of the concrete surface.

**Figure 5:**
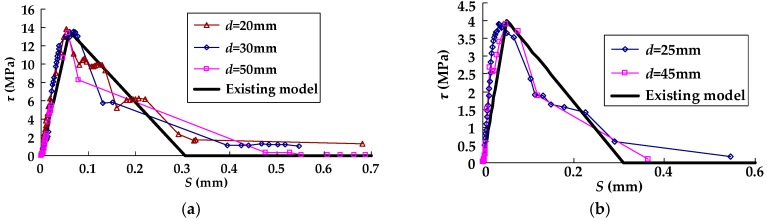

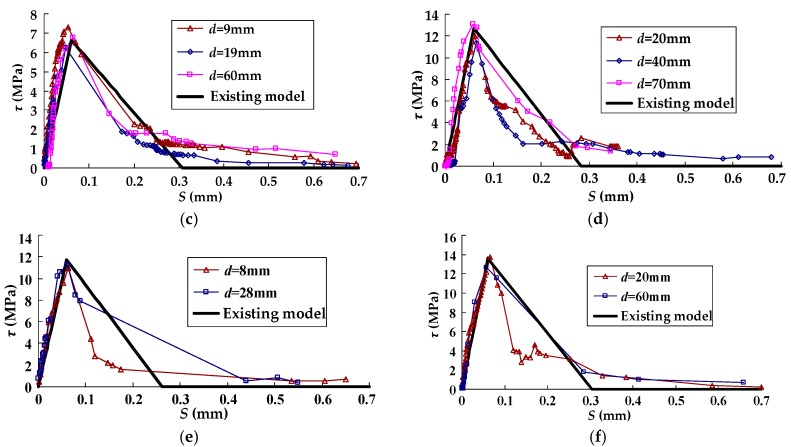
The comparison between the existing bond–slip model and test results for specimens: (**a**) A-0; (**b**) B-01; (**c**) B-02; (**d**) C-01; (**e**) C-02; and (**f**) D-0.

The test results of the bond–slip curves at two or three points (the distance between the point and the bond end of the CFRP sheet near the midspan is represented by *d*) of the CFRP–concrete interface for each static specimen in groups A, B, C, and D were compared with this bond–slip model (seen in Figure 5). As shown in Figure 5, the test results agreed well with the bond–slip model, which indicate that this model can be accepted to predict the bond stress–slip relationship under static loading. However, this model can not be used to describe the bond stress–slip relationship under cyclic loading, because the degradation of the bond stress–slip curve can not be considered in this model. So, a modified bond–slip model for predicting the bond stress–slip relationship of the CFRP–concrete interface under cyclic loading was analyzed below, as based on test results.

### 3.2. Bond–Slip Model under Cyclic Loading

It has been demonstrated that the interfacial fracture energy (*G_f_*) remains unchanged after suffering cyclic loading when the residual bond length is longer than the effective bond length [25]. In addition, the local slip corresponding to the maximum shear stress (*S*_0_) can be seen to be constant during cyclic loading, according to the above research. Thus, based on the bond–slip model under the static loading presented above, the bond–slip model of the CFRP–concrete interface under cyclic loading can be expressed as follows:

(9) $$\tau =\left\{ \begin{array}{cc} \frac{\tau_{ft}}{S_{0}}S & 0\leq S\leq S_{0}\\ \frac{\tau_{ft}}{S_{ft}-S_{0}}(S_{ft}-S) & S_{0}<S\leq S_{ft}\\ \tau =0 & S>S_{ft} \end{array}\right.$$

where:

(10) $$S_{ft}=\frac{2G_{f}}{\tau_{ft}}=\frac{\tau_{f0}S_{f0}}{\tau_{ft}}$$

In Equations (9) and (10), *τ_ft_* is the maximum shear stress, as varied with the number of load cycles, *S_ft_* indicates the demarcation value of slip between the descent and horizontal segments of the bond–slip curve, which varied with the number of load cycles, *τ_f_*_0_ (which is equal to *τ*_max_) denotes the maximum shear stress when the CFRP–concrete interface is subjected to the initial loading, and *S_f_*_0_ represents the demarcation value of slip between the descent and horizontal segments of the bond–slip curve under the initial loading.

### 3.3. Degradation Law of τ_ft_

**Figure 6:**
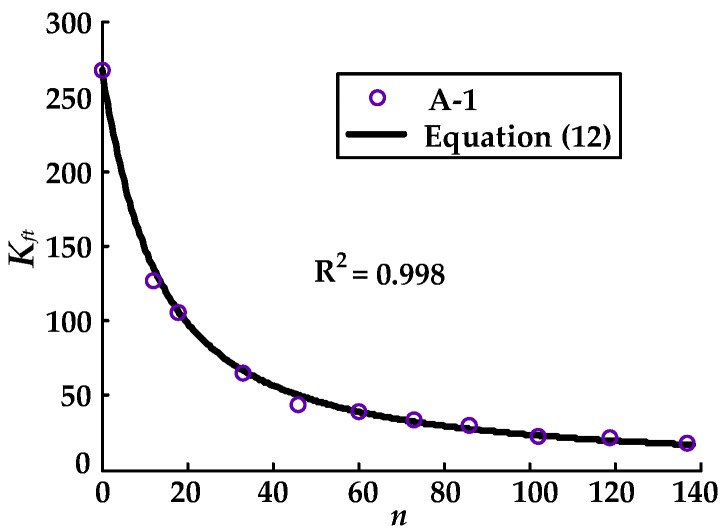
The relationship between *K_ft_* and load cycles (*n*).

The parameter *τ_ft_* can be expressed by:

(11) $$\tau_{ft}=K_{ft}S_{0}$$

where *K_ft_* represents the slope of the ascending segment of the bond–slip curve as varied with the number of load cycles. Since *S*_0_ is constant, the degradation law of *K_ft_* is discussed below in order to capture the degradation law of *τ_ft_*. The degradation law of *K_ft_* can be detected from the test results of the bond–slip curves. The degradation of *K_ft_* with load cycles (*n*) obtained according to the test results for specimen A-1 is presented in Figure 6. As shown in Figure 6, *K_ft_* decreased nonlinearly with the number of load cycles, and the rate of decline in *K_ft_* decreased with the number of load cycles. This phenomenon was typical for the other specimens. By regression analysis of the test results, the relationship between *K_ft_* and the load cycles (*n*) can be expressed by the following equation:

(12) $$K_{ft}=\frac{\tau_{ft}}{S_{0}}=\frac{K_{0}}{1+cn^{b}}$$

where *K*_0_, which is equal to *τ_f0_*/*S*_0_, which represents the slope of the ascending segment of the bond–slip curve at the initial loading (the corresponding number of load cycles, *n*, is 0), and *c* and *b* are the coefficients as determined by the test results. It can be seen from Figure 6 that Equation (12) is in good agreement with the test results, with a correlation coefficient (*R*^2^) of 0.998. The concrete cube strength (*f*_cu_), cyclic load level (*S_c_*), CFRP-to-concrete width ratio (*b_f_*/*b*_c_), bond length of the CFRP sheets (*l_f_*) and values of *c* and *b* obtained by a regression analysis of the test results for each cyclic specimen in groups A, B, C, and D are listed in Table 2. The load level (*S_c_*) applied on each cyclic specimen was calculated by:

(13) $$S_{c}=\frac{\Delta S}{1-S_{a}}$$

where

(14) $$S_{a}=\frac{P_{\max}+P_{\min}}{2P_{u}}$$

As shown in Table 2, a comparison of specimens A-1, A-2, A-3, and A-4 indicates that the values of both *c* and *b* increased with an increase in the load level (*S_c_*). It can be seen by comparing specimens A-3, B-1, and B-2 that the value of *b* decreased with an increase in concrete strength, while the change of concrete strength had little effect on the value of *c*. A comparison of specimens A-2, C-1, and C-2 showed that the value of *b* increased as the CFRP-to-concrete width ratio increased, while the value of *c* remained constant as the CFRP-to-concrete width ratio varied. A comparison between specimens A-2 and D-1 indicated that the values of *c* and *b* were almost constant as the bond length increased. These phenomena reveals that the rate of reduction in the slope of the ascending segment of the bond–slip curve (*K_ft_*) increased as the load level (*S_c_*) or CFRP-to-concrete width ratio enhanced, but decreased with an increase in concrete strength. In addition, the bond length had little effect on the rate of reduction in the slope of the ascending segment of the bond–slip curve (*K_ft_*) under cyclic loading on the condition that the residual bond length was longer than the effective bond length. By a regression analysis of the relationship between the fitting results of *c* and the load levels (*S_c_*) for specimens A-1, A-2, A-3, and A-4, the coefficient *c* can be given by the following equation (with a correlation coefficient of 0.999):

(15) $$c\;=0.0007e^{2.919S_{c}{}^{2}}$$

Using a regression analysis of the relationship between the values of *b* and the load levels (*S_c_*) for specimens A-1, A-2, A-3, and A-4, a linear relationship can be found between *b* and *S_c_* (with a correlation coefficient of 0.987):

(16) $$b=0.873S_{c}+0.0198$$

Considering the fitting results of *b* for specimens A-3, B-1, and B-2, regression analysis revealed the following linear relationship between *b*/*b*_A-3_ and concrete strength (*f_cu_*) (with a correlation coefficient of 0.982):

(17) $$\frac{b}{b_{A-3}}=1.208-0.00337f_{cu}$$

Based on a regression analysis of the fitting results of *b* for specimens A-2, C-1, and C-2, a linear relationship between *b*/*b*_A-2_ and a CFRP-to-concrete width ratio (*b_f_/b_c_*) was found and expressed by the following equation (with a correlation coefficient of 0.982):

(18) $$\frac{b}{b_{A-2}}=0.619\frac{b_{f}}{b_{c}}+0.838$$

Making *b* in Equation (16) equal to *b*_A-3_, and substituting Equation (16) into Equation (17), *b* can be calculated by the following equation, which considers the effects of load level (*S_c_*) and concrete strength (*f_cu_*):

(19) $$b=(0.873S_{c}+0.0198)(1.208-0.00337f_{cu})$$

Setting *b* in Equation (19) as equal to *b*_A-2_, and substituting Equation (19) into Equation (18), the calculation equation of *b*, which considers the influences of load level (*S_c_*), concrete strength (*f_cu_*), and the CFRP-to-concrete width ratio (*b_f_/b_c_*), can be given as follows:

(20) $$b=(0.873S_{c}+0.0198)(1.208-0.00337f_{cu})(0.619\frac{b_{f}}{b_{c}}+0.838)$$

### 3.4. Verification of Proposed Model

**Figure 7:**
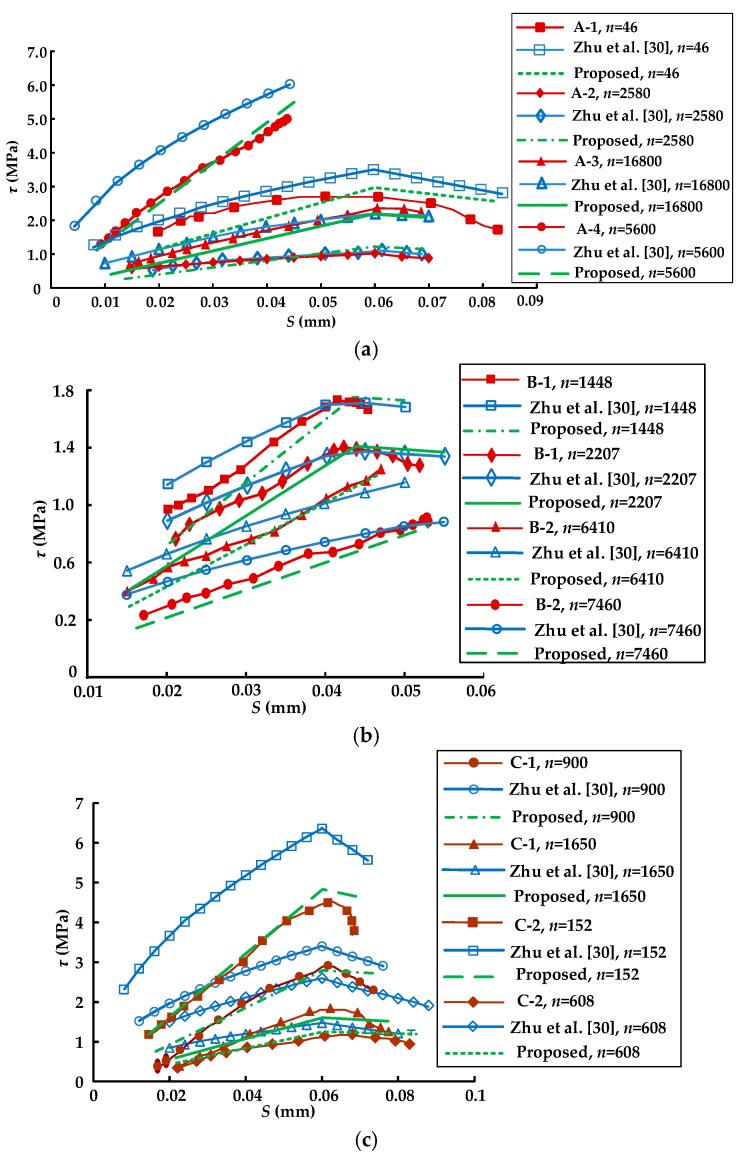
The comparison of the existing model [30], the proposed model, and the test results of the specimens in groups: (**a**) A; (**b**) B; and (**c**) C.

**Figure 8:**
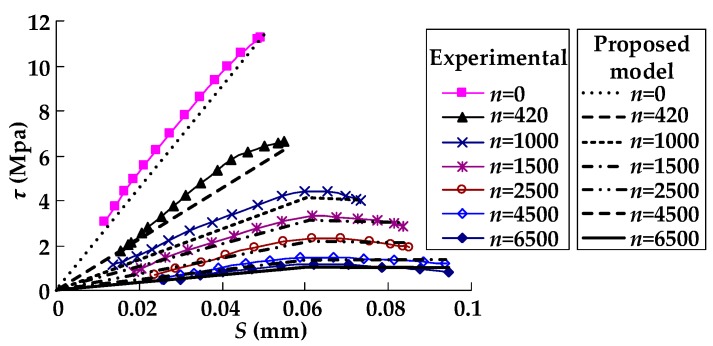
The comparison between the proposed model and the test results of specimen D-1.

The calculated results of the bond–slip curves at different numbers of load cycles (*n*) using the proposed model (expressed by Equations (9)–(12), (15) and (20)) and the bond–slip model suggested by Zhu et al. [30] were compared with the test results for the specimens in groups A, B, and C (as seen in Figure 7). As shown in Figure 7a,b, both the proposed model in this paper and the model suggested by Zhu et al. [30] well captured the overall trend of the bond–slip relationship of the test specimens in groups A and B. However, it can be seen from Figure 7c that the proposed model in this paper agreed well overall with the test results, while the model suggested by Zhu et al. [30] was not in good agreement with test results of specimens C-1 and C-2. The reason for this phenomenon may be that the model suggested by Zhu et al. [30] did not consider the effect of the CFRP-to-concrete width ratio on the fatigue property of the bond between the CFRP sheets and the concrete. In addition, the bilinear model proposed in this paper can be simpler and more efficient compared with the nonlinear model suggested by Zhu et al. [30] when it is used in analytical or numerical calculation. Figure 8 presents the comparison between the calculated results of the bond–slip curves at different numbers of load cycles (*n*) using the proposed model (expressed by Equations (9)–(12), (15), and (20)) and the test results for specimen D-1. As shown in Figure 8, the calculated results agreed well overall with the test results. The above analysis indicates that the proposed bond–slip model can be used to describe the bond–slip relationship between the CFRP sheet and concrete under cyclic loading.

## 4. Conclusions

In this paper, experimental and theoretical research was conducted in order to investigate the bond–slip relationship between CFRP sheets and concrete under cyclic loading. Two variables, namely, the CFRP-to-concrete width ratio and the bond length of the CFRP sheets, were considered in this experimental research through using modified beam specimens subjected to static and cyclic loading. A bilinear bond–slip model was developed to describe the bond–slip relationship of the CFRP–concrete interface under cyclic loading based on the existing bond–slip model for the CFRP–concrete interface under static loading, the test results presented in this paper, and the existing test results. The following conclusions can be drawn according to investigations in this paper:

(1) By comparing with the test results, the bond–slip model (expressed by Equations (3)–(8)) was verified as an acceptable prediction of the bond stress–slip relationship under static loading.
(2) The slope of the ascending segment of the bond–slip curve decreased with an increase in the number of load cycles, but the slip corresponding to the maximum bond shear stress was almost unchanged as the number of load cycles increased. The rate of reduction in the slope of the ascending segment of the bond–slip curve during cyclic loading increased with an increase in the load level or the CFRP-to-concrete width ratio, but decreased with an increase in concrete strength. The bond length had little effect on the rate of reduction in the slope of the ascending segment of the bond–slip curve under cyclic loading if the residual bond length was longer than the effective bond length.
(3) A good agreement between the developed bilinear bond–slip model and test results indicates that the proposed model in this paper can be applied to predict the bond–slip relationship between the CFRP sheets and concrete under cyclic loading.

## Figures and Tables

**Table 1:** Details of specimens and test results.

Group	Specimen	Test Method	∆*S* ^1^	*P*_max_ (kN)	∆*P* (kN)	*f_cu_* (MPa)	*b_f_*/*b_c_*	*l_f_* (mm)	*N* (Cycles)
A	A-0	Static	–	29.17	–	62.2	0.25	160	1
	A-1	cyclic	0.65	23.34	18.96	62.2	0.25	160	2600
	A-2	cyclic	0.55	20.42	16.04	62.2	0.25	160	32,000
	A-3	cyclic	0.5	18.96	14.58	62.2	0.25	160	168,900
	A-4	cyclic	0.4	16.04	11.67	62.2	0.25	160	1,550,000
B	B-01	Static	–	12.98	–	25.1	0.25	160	1
	B-1	cyclic	0.5	8.44	6.49	25.1	0.25	160	67,000
	B-02	Static	—	21.30	—	35.3	0.25	160	1
	B-2	cyclic	0.5	13.85	10.65	35.3	0.25	160	88,300
C	C-01	Static	–	42.85	–	62.2	0.35	160	1
	C-1	cyclic	0.55	30.00	23.57	62.2	0.35	160	20,680
	C-02	Static	–	47.64	–	62.2	0.5	160	1
	C-2	cyclic	0.55	33.35	26.20	62.2	0.5	160	11,830
D	D-0	Static	–	30.18	–	62.2	0.25	240	1
	D-1	cyclic	0.55	21.13	16.60	62.2	0.25	240	90,000

^1^ ∆*S* = (*P*_max_ − *P*_min_)/*P_u_*.

**Table 2:** Fitting results of *c* and *b*.

Specimen	A-1	A-2	A-3	A-4	B-1	B-2	C-1	C-2	D-1
*f_cu_* (MPa)	62.2	62.2	62.2	62.2	25.1	35.3	62.2	62.2	62.2
*S_c_*	1.238	0.957	0.833	0.615	0.833	0.833	0.957	0.957	0.957
*b_f_*/*b_c_*	0.25	0.25	0.25	0.25	0.25	0.25	0.35	0.5	0.25
*l_f_* (mm)	160	160	160	160	160	160	160	160	240
*c*	0.0609	0.0105	0.00562	0.00205	0.00565	0.00563	0.0105	0.0105	0.0104
*b*	1.115	0.851	0.712	0.581	0.802	0.773	0.870	0.981	0.853
*R*^2^ *	0.998	0.998	0.994	0.983	0.994	0.992	0.997	0.976	0.995

* *R*^2^ denotes the correlation coefficient between the experimental results and predicted results using Equation (12) with coefficients (*c* and *b*) of the values in Table 2 for each specimen.
